# Multi-sequence MRI signal intensity ratios as candidate imaging correlates for audiovestibular dysfunction in vestibular schwannoma

**DOI:** 10.3389/fneur.2026.1936823

**Published:** 2026-09-23

**Authors:** Yun Li, Xuening Zhao, Xianshun Yuan, Peng Zhao, Xiaoyan Dai, Yingjuan Ma, Yiyuan Zhu, Gang Chen

**Affiliations:** 1 Department of Otolaryngology Head and Neck Surgery, Shandong Provincial Hospital Affiliated to Shandong First Medical University, Jinan, China; 2 Department of Radiology, Shandong Provincial Hospital Affiliated to Shandong First Medical University, Jinan, China

**Keywords:** 3D-FLAIR, audiovestibular dysfunction, MRI, T2WI, vestibular schwannoma

## Abstract

**Introduction:**

Vestibular schwannoma (VS) typically induces gradual sensorineural hearing impairment and vestibular dysfunction. Nevertheless, clinically applicable non-invasive candidate imaging markers capable of reflecting pathological alterations within the inner ear have not yet been well established. The present study sought to systematically investigate the correlative relationships between labyrinth signal intensity ratios acquired from multi-sequence inner ear magnetic resonance imaging (MRI) and audiovestibular functional deficits in patients diagnosed with VS.

**Methods:**

A total of 22 patients with radiologically confirmed VS were consecutively enrolled for this retrospective observational study. All subjects received 3.0 T inner ear MRI examinations covering three pulse sequences: T2-weighted imaging (T2WI), pre-contrast three-dimensional fluid-attenuated inversion recovery (3D-FLAIR), and delayed post-contrast 3D-FLAIR acquired at the 4-h time point after gadolinium administration. Signal intensity ratios (cochlea-, vestibule-, and semicircular canal-to-brainstem; CBR, VBR, SBR) were measured and correlated with pure-tone audiometry, caloric testing, and video head impulse test (vHIT) results.

**Results:**

Compared to contralateral unaffected ears, affected ears exhibited significantly elevated signal ratios on both delayed post-contrast and pre-contrast 3D-FLAIR sequences (all *p* < 0.001), indicating blood-labyrinth barrier disruption, while T2WI ratios were significantly reduced (*p* < 0.001), suggesting structural alterations like increased protein content. Correlation analysis revealed that pre-contrast 3D-FLAIR CBR exhibited a moderate positive correlation with the degree of hearing loss (*r* = 0.582, *p* = 0.037), whereas T2WI SBR showed a correlation with low-frequency vestibular dysfunction assessed by caloric testing (*r* = 0.440, *p* = 0.040). In contrast, none of the evaluated MRI metrics demonstrated significant correlations with high-frequency vestibular function measured by vHIT.

**Conclusion:**

These findings demonstrate that specific inner ear MRI signal ratios serve as promising candidate imaging correlates for auditory impairment and low-frequency vestibular deficits in VS. Pre-contrast 3D-FLAIR and T2WI sequences offer distinct insights into inflammatory leakage and structural damage, respectively. The potential for these metrics to guide personalized management strategies remains to be evaluated in future studies. Large-scale multicenter investigations incorporating histopathologic verification are therefore needed to dissect these underlying mechanisms and develop predictive prognostic frameworks.

## 1 Introduction

Vestibular schwannoma (VS) is histologically benign yet markedly compromises patients’ quality of life by triggering progressive sensorineural hearing loss and vestibular dysfunction, which also creates a heavy socioeconomic healthcare burden (1). Existing therapeutic regimens, ranging from microsurgical resection and stereotactic radiosurgery to conservative surveillance, all entail distinct risks of irreversible auditory and vestibular damage. The absence of reliable candidate imaging markers to forecast functional deterioration further hinders individualized clinical risk stratification and treatment planning (2). Consequently, there is a pressing need for novel non-invasive imaging correlates to assess inner ear pathology and guide personalized treatment in VS.

High-resolution magnetic resonance imaging (MRI) has emerged as a powerful non-invasive tool for visualizing pathological alterations within the inner ear labyrinth (3). In VS research, delayed post-contrast three-dimensional fluid-attenuated inversion recovery (3D-FLAIR) sequences have shown considerable diagnostic value. Elevated signal intensity within the tumor-impacted labyrinth is consistently detected on post-contrast 3D-FLAIR images, a hallmark imaging feature that reflects compromised integrity of the blood-labyrinth barrier (4–6). Meanwhile, the present study also demonstrates that this asymmetric enhancement correlates significantly with the degree of hearing loss, establishing a direct link between an imaging metric and a functional symptom (4, 5). Beyond post-contrast changes, alterations on pre-contrast 3D-FLAIR sequences have also been noted (7–11). In addition, T2-weighted (T2WI) signal intensity, which reflects the fluid content of the labyrinth, has been explored as a predictor of hearing outcomes, with degraded pre-operative cochlear T2 signal correlating with more severe postoperative hearing loss after tumor resection (12). Taken together, these results suggest that multiparametric MRI can act as a non-invasive surrogate for evaluating inner ear functional status, analogous to an *in vivo* virtual biopsy.

Despite these encouraging advances, a systematic understanding of the integrated information provided by different MRI sequences in the context of VS remains elusive. A significant limitation of the existing literature is that most studies have investigated individual MRI contrasts in isolation, primarily focusing on pre-contrast 3D-FLAIR plain or immediate post-contrast 3D-FLAIR sequence (4, 6). The simultaneous, comparative evaluation of how T2WI, pre-contrast 3D-FLAIR, and delayed post-contrast 3D-FLAIR sequences collectively depict the pathological landscape of the tumor-affected inner ear in the same patient cohort has not been thoroughly performed. Furthermore, the correlation between these imaging readouts and comprehensive audiovestibular function is largely restricted to basic audiological measures (7, 8, 11), often neglecting a detailed assessment of the vestibular end-organ. Lee et al. reported no correlation between pre-contrast 3D-FLAIR signal intensity of the vestibule and vestibular function test outcomes (9). To date, however, no studies have explored associations between labyrinthine signal hyperintensity on T2WI or delayed post-contrast 3D-FLAIR sequences and dedicated vestibular function assessments, including caloric irrigation and the video head impulse test (vHIT). The interplay between findings across multiple MRI sequences and vestibular function therefore constitutes a key unresolved area for further research.

This study combines quantitative data from multiple MRI sequences with complete audiological and vestibular function tests in a systematic manner. By pairing imaging measurements with functional test results, we aimed to verify whether these non-invasive MRI correlates can accurately predict distinct patterns of hearing and vestibular damage in patients with VS. The findings may inform future work exploring whether these MRI metrics could support individualized treatment strategies.

## 2 Materials and methods

### 2.1 Study subjects

This retrospective observational study consecutively enrolled eligible patients with VS at Shandong Provincial Hospital Affiliated to Shandong First Medical University between November 2021 and February 2026. Inclusion criteria: (1) patients aged ≥18 years; (2) definitive diagnosis of VS verified by inner ear magnetic resonance imaging; (3) complete baseline clinical data available, including pure tone audiometry, caloric testing, and vHIT. Exclusion criteria: (1) prior surgical or radiosurgical intervention for VS; (2) history of other major otological disorders or previous otologic surgery that could confound audiovestibular functional assessments (e.g., chronic suppurative otitis media); (3) incomplete key audiovestibular or imaging variables required for outcome analysis. A total of 22 patients met all criteria and were included in the final analysis. Patient screening was documented in the flow diagram (Figure 1). All raw data in this study were sourced from the hospital inpatient system, and anonymization and desensitization processing for all personnel data had been completed. The study protocol was approved by the Ethics Review Committee of the Shandong Provincial Hospital Affiliated to Shandong First Medical University (NO. 2026-560).

**Figure 1:**
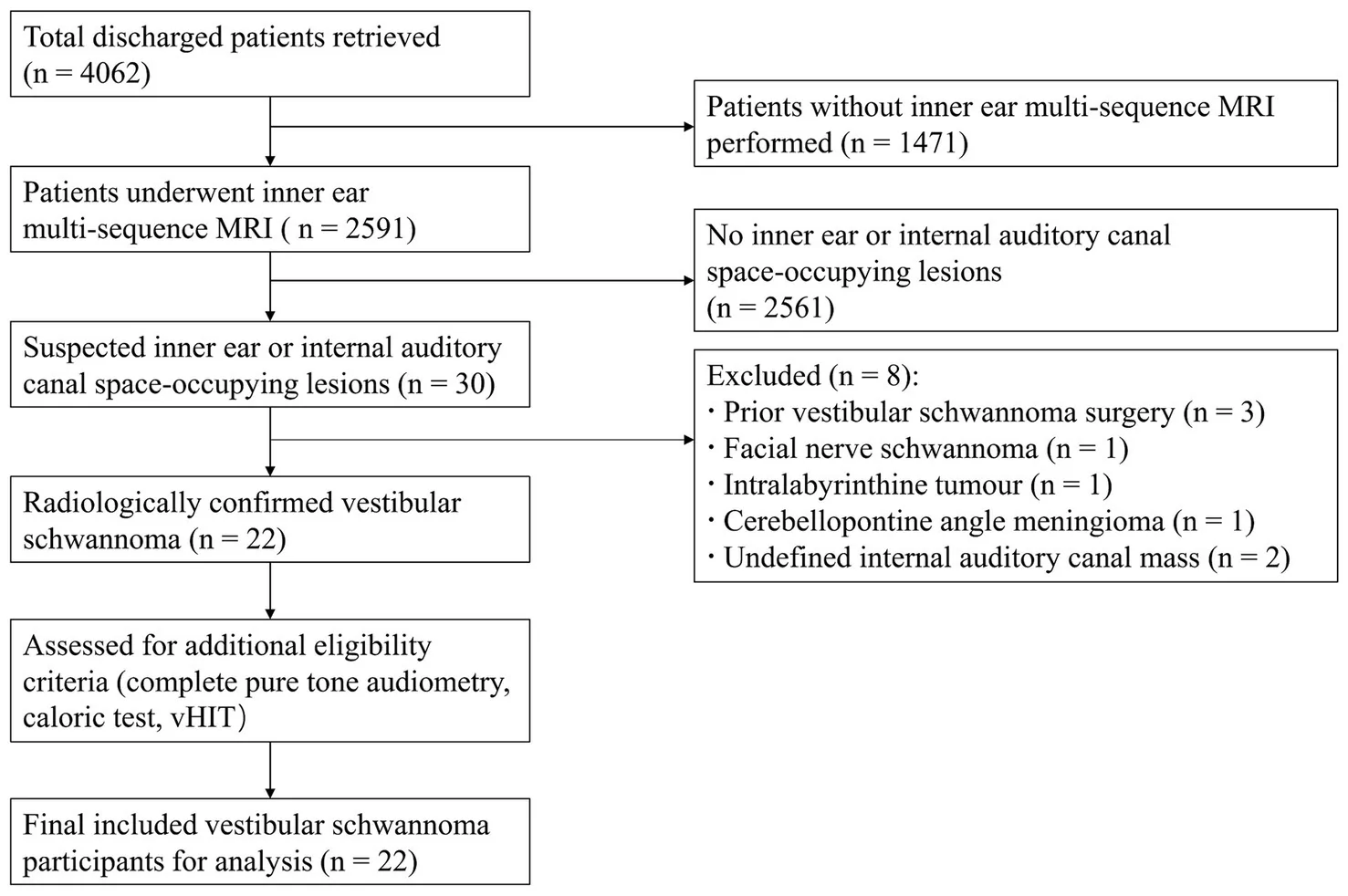
Patients screening flow chart.

### 2.2 Magnetic resonance imaging examination protocol

All subjects underwent inner ear MRI scanning using a 3.0 T magnetic resonance scanner (MAGNETOM Prisma, Siemens, Germany) combined with a dedicated 64-channel head coil. The scanning sequences and specific parameter settings were as follows:

Inner ear small field-of-view three-dimensional variable flip angle fast spin echo T2WI: repetition time (TR) 1,000 ms, echo time (TE) 126 ms, field of view (FOV) 210 mm, isotropic voxel 0.3 × 0.3 × 0.3 mm, single sequence acquisition duration 6 min 18 s (Figure 2A).

**Figure 2:**
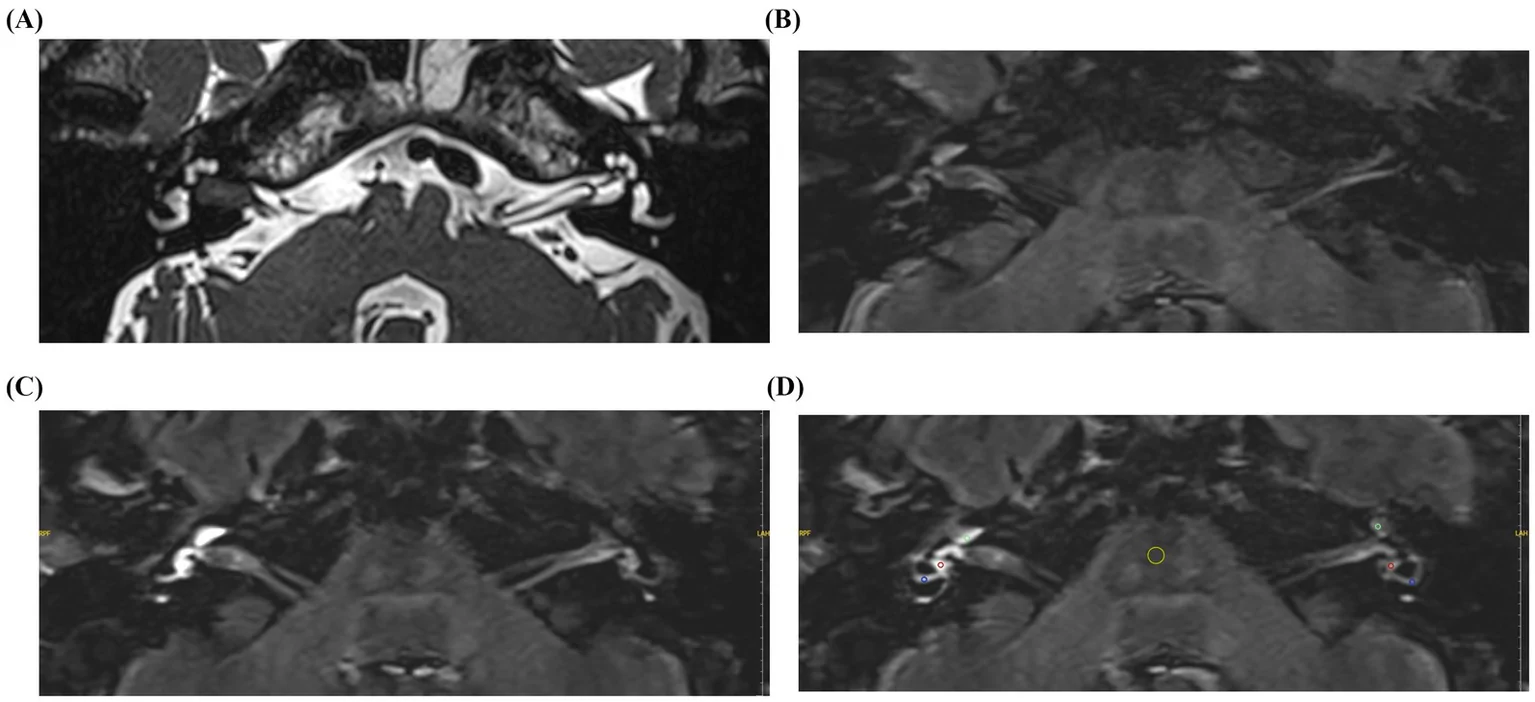
Axial inner ear MRI from a patient with a right‑sided internal auditory canal vestibular schwannoma. **(A)** Axial T2‑weighted imaging (T2WI) demonstrates the hypointense lesion in the right internal auditory canal, with reduced signal intensity in the right cochlea and vestibule relative to the left side. **(B)** Pre‑contrast 3D‑FLAIR shows isointense signal of the right inner ear compared with the contralateral left inner ear. **(C)** Delayed post‑contrast 3D‑FLAIR reveals hyperintense signal within the right inner ear relative to the left side. **(D)** On delayed post‑contrast 3D‑FLAIR imaging, small 1 mm² regions of interest (ROIs) were manually placed in the basal turn of the cochlea, vestibule, and horizontal semicircular canal; a 10 mm² control ROI was positioned within the brainstem parenchyma for signal‑intensity ratio calculation.

Inner ear axial 3D-FLAIR: TE 280 ms, TR 8000 ms, inversion time (IR) 2,250 ms, FOV 160 mm, voxel 0.3 × 0.3 × 1 mm, single sequence acquisition duration 12 min 56 s (Figure 2B).

All patients received intravenous injection of double-dose contrast agent Magnevist (gadopentetate dimeglumine) at a dosage of 0.4 mL/kg; 4 h after injection completion, delayed enhancement scanning was performed using the 3D-FLAIR sequence completely consistent with the plain scan (Figure 2C).

### 2.3 Image post-processing and analysis

All image analysis work was completed relying on image post-processing workstation (GE HealthCare Centricity RIS CE, United States). Regions of interest (ROI) were delineated, respectively, in three groups of sequence images: T2WI, pre-contrast 3D-FLAIR plain scan, and 4-h delayed enhancement 3D-FLAIR post-contrast agent injection: small ROIs of 1 mm^2^ were set in the basal turn or middle turn of the cochlea, vestibule, and horizontal semicircular canal regions, and a large control ROI of 10 mm^2^ was set in the brainstem parenchyma region (Figure 2D). The cochlea-brainstem ratio (CBR), vestibule-brainstem ratio (VBR), and semicircular canal-brainstem ratio (SBR) were calculated synchronously. ROIs were manually drawn on matched inner ear anatomical structures for both the tumor-affected and contralateral unaffected ears. In this manuscript, the term “unaffected” refers to inner ear structures that are not anatomically involved by the vestibular schwannoma, and does not imply complete physiological normality. The contralateral unaffected labyrinth served as a subject matched internal control for each patient, to mitigate inter-individual MRI signal variations.

The ROI delineation and signal value measurement for all imaging images were independently completed by one senior radiologist (P. Z.) with 20 years of experience in central nervous system and inner ear imaging diagnosis; this physician was completely blinded to clinical outcome data such as hearing and vestibular function for all patients to avoid subjective measurement bias.

### 2.4 Audiometric and vestibular tests

All participants underwent pure tone audiometry within a sound-isolated testing booth via an audiometer (Astera, Otometrics, Denmark). Air-conduction thresholds were recorded across frequencies ranging from 125 Hz to 8,000 Hz, including 125, 250, 500, 1,000, 2000, 4,000 and 8,000 Hz. The four-frequency pure tone average was subsequently computed based on threshold values measured at 500, 1000, 2000 and 4,000 Hz, if there was no auditory response at a single frequency detection, the hearing threshold for that frequency was uniformly assigned a value of 110 dB HL for statistical purposes.

The bithermal caloric test evaluated ultra-low-frequency vestibular function of the bilateral horizontal semicircular canals, which was routinely performed with videonystagmography instrument (ICS Chartr 200, Otometrics, Denmark). A semicircular canal paresis value >25% was defined as unilateral abnormality. Bilateral canal paresis was diagnosed as a combined slow-phase velocity (SPV) ≤ 12°/s across bithermal caloric irrigation (13). The semicircular canal paresis value was calculated according to standard Jongkees’ formula: Canal paresis (%) = [(RW + RC) − (LW + LC)] / (RW + RC + LW + LC) × 100% (RW: right ear warm SPV; RC: right ear cold SPV; LW: left ear warm SPV; LC: left ear cold SPV). Caloric test outcomes were classified as normal or abnormal for statistical analyses.

The vHIT evaluated high-frequency vestibular function of the six semicircular canals, which was evaluated using available video oculography system (ICS impulse 2.0, Otometrics, Denmark). vHIT results were defined as pathological only when the gain reduction (vertical canal gain < 0.7, horizontal canal gain < 0.8) and reproducible catch-up saccades were both present for at least one semicircular canal (13). Isolated gain reduction without catch-up saccades or catch-up saccades with normal gain was not classified as pathological.

### 2.5 Statistical analysis

All statistical calculations were carried out with SPSS version 31.0. Continuous variables were presented as mean ± standard deviation. For intra-individual comparisons between the lesioned and contralateral intact ear, paired *t*-tests were adopted for normally distributed datasets, whereas non-normally distributed parameters were assessed via the Wilcoxon signed-rank test. The normality of continuous variables was evaluated using the Shapiro–Wilk test combined with Q-Q plots. Pure-tone average hearing thresholds and subsets of MRI signal intensity ratios deviated from normal distribution. Therefore, the non-parametric Spearman’s rank correlation test was adopted for exploratory associations between quantitative labyrinthine MRI signal ratios and auditory functional metrics. Pearson correlation or Mann–Whitney U test were used to analyze the associations between quantitative labyrinthine MRI signal ratios and bithermal caloric test as well as vHIT outcomes. Statistical significance was set at a threshold of *p* < 0.05.

This retrospective single center imaging study is an exploratory study, and no single main outcome index is set in advance. Two sets of analyses were distinguished in the present work. Main analyses of interest included inter-aural comparisons of labyrinth signal-to-brainstem ratios across three MRI sequences between VS-affected and contralateral unaffected ears. All correlation analyses between MRI metrics and audiovestibular functional outcomes were regarded as exploratory analyses.

## 3 Result

### 3.1 Baseline clinical and tumor characteristics

Twenty-two patients diagnosed with VS were enrolled in the present cohort. Their demographic profiles and tumor features were summarized in Table 1. The study population consisted of 6 male and 16 female participants, with ages spanning 32 to 73 years and a mean age of 56.0 ± 12.0 years. All 22 patients presented with complaints of dizziness (100%), with disease duration spanning from 1 d to 17 years; among them, 19 cases (86.4%) were accompanied by hearing loss, and 14 cases (63.6%) had concurrent tinnitus symptoms. Tumors arose on the left side in 13 patients and the right side in the remaining 9 participants. The maximal tumor diameter ranged from 3 mm to 18 mm, with a mean value of 8.96 ± 4.08 mm. All lesions were stratified by the Koos grading scale: 11 tumors were classified as Grade I, 10 as Grade II, 1 as Grade III, and none reached Grade IV. All patients completed pure-tone audiometry, caloric testing, and the full set of vHIT vestibular function tests synchronously within 3 days prior to MRI examination.

**Table 1:** Clinical manifestations and audiovestibular test profiles in patients with vestibular schwannoma.

Age (years, mean±SD)	56.0 ± 12.0
Men:Women	6:16
Right:Left	9:13
Presence of dizziness, *n* (%)	22 (100%)
Presence of hearing loss, *n* (%)	19 (86.4%)
Presence of tinnitus, *n* (%)	14 (63.6%)
Initial PTA (dB)	53.4 ± 24.9
Caloric testing abnormal, *n* (%)	16 (72.7%)
vHIT abnormal, *n* (%)	6 (27.3%)
Tumor size (mm)	8.96 ± 4.08
Koos classification	
I, Pure intracanalicular tumor, *n* (%)	11 (50.0%)
II, Small CPA mass (~2 cm), brainstem untouched, *n* (%)	10 (45.5%)
III, Full CPA occupancy (~3 cm), brainstem contact, no shift, *n* (%)	1 (4.5%)
IV, Large tumor displacing brainstem and cranial nerves, *n* (%)	0 (0%)

PTA, pure-tone audiolometry; vHIT, video head impulse test; CPA, cerebellopontine angle; *n*, number of patients.

Nine patients lacked pre-contrast 3D-FLAIR sequences: five patients did not undergo this sequence due to incomplete institutional MRI protocols between November 2021 and June 2022, and the other four patients were admitted with a provisional diagnosis of Ménière’s disease and received only T2WI and 4-h delayed post-contrast 3D-FLAIR for endolymphatic hydrops assessment. The remaining 13 subjects completed all three sequences: T2WI, pre-contrast 3D-FLAIR, and 4-h delayed post-contrast 3D-FLAIR. One patient (X. J) had moderate vestibular endolymphatic hydrops on the affected side; this case was excluded in the intergroup comparative analysis of VBR.

### 3.2 Audiological and vestibular function test results

The pure-tone audiometry threshold of the affected ear was 16.25 ~ 110 dB HL, with a mean of (53.4 ± 24.9) dB HL; the audiometry threshold of the contralateral unaffected ear was 10 ~ 86.25 dB HL, with a mean of (29.0 ± 19.5) dB HL. Affected ears exhibited markedly poorer hearing thresholds relative to contralateral unaffected ears, with the interaural difference reaching statistical significance via paired comparison (*p* < 0.001). Caloric testing revealed abnormal responses in 16 out of 22 patients (72.7%). Among these abnormal cases, 12 patients presented with unilateral canal paresis of the tumor-affected ear, and 4 patients demonstrated bilateral canal paresis. The remaining 6 patients had normal caloric test results. For patients with unilateral weakness, the canal paresis values ranged from 28 to 89%. On vHIT, 6 of the 22 patients (27.3%) exhibited pathological vHIT findings ipsilateral to the tumor, all of whom demonstrated reproducible catch-up saccades. Specifically, four patients presented reduced gain of the horizontal semicircular canal with catch-up saccades; one patient had gain reduction across all three ipsilateral semicircular canals accompanied by catch-up saccades; and one patient showed decreased gain in both the horizontal and posterior semicircular canals, with catch-up saccades confined to the horizontal semicircular canal. All principal audiometric and vestibular function test outcomes were summarized in Table 1.

### 3.3 Inner ear MRI signal ratios between VS-affected and contralateral unaffected ears

For delayed post-contrast 3D-FLAIR sequences, CBR, VBR and SBR values were substantially elevated in the tumor-affected labyrinth compared with the intact contralateral side; all interaural differences achieved statistical significance (all *p* < 0.001) (Figure 3). Specific values: affected side CBR (5.00 ± 1.66), unaffected side CBR (2.19 ± 0.96); affected side VBR (5.09 ± 2.07), unaffected side VBR (2.77 ± 1.19); affected side SBR (2.03 ± 0.80), unaffected side SBR (1.26 ± 0.56).

**Figure 3:**
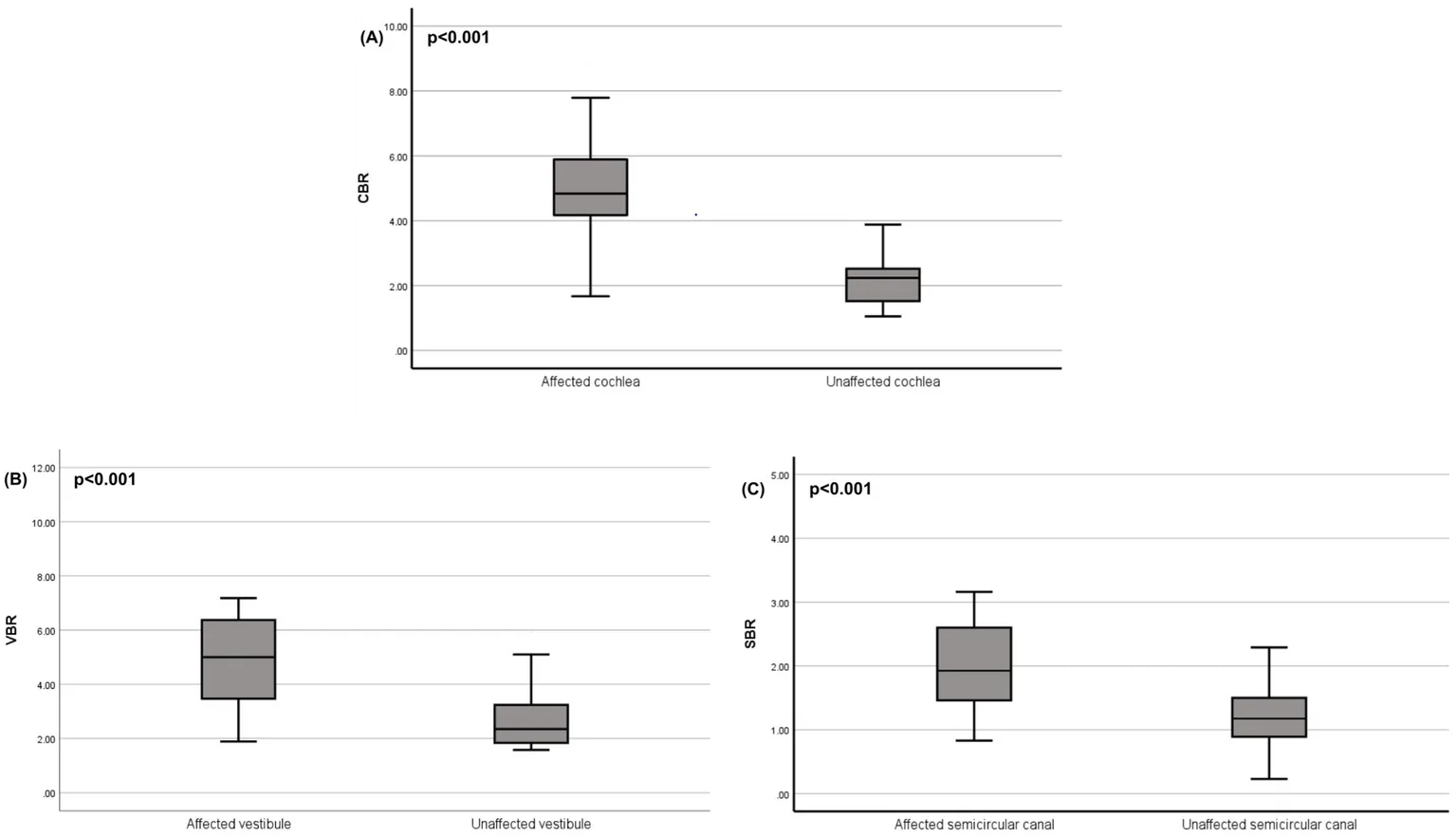
Quantitative signal ratios on delayed post-contrast 3D-FLAIR imaging. Significantly higher CBR **(A)**, VBR **(B)**, and SBR **(C)** values were observed in the tumor-affected labyrinth relative to the unaffected contralateral labyrinth, with all interaural differences statistically significant (all *p* < 0.001).

On T2WI scans, all three inner ear signal ratios were markedly reduced in the tumor-bearing ear relative to the contralateral normal ear, with every interaural comparison yielding statistical significance (all *p* < 0.001) (Figure 4). Specific values: affected side CBR (3.51 ± 0.80), unaffected side CBR (4.71 ± 0.94); affected side VBR (4.08 ± 0.71), unaffected side VBR (4.91 ± 0.58); affected side SBR (3.34 ± 0.74), unaffected side SBR (4.11 ± 0.86).

**Figure 4:**
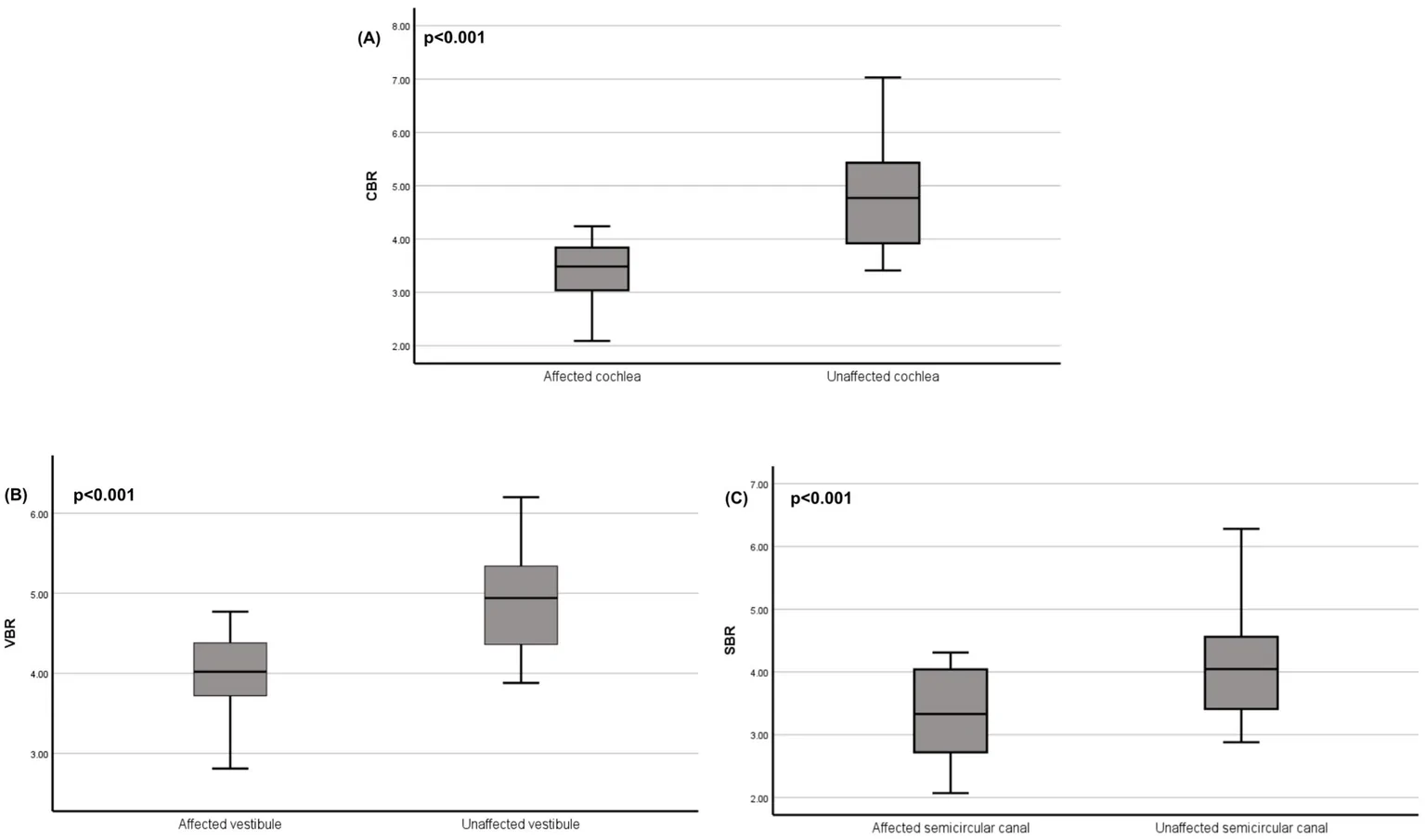
Quantitative signal ratios on T2WI. CBR **(A)**, VBR **(B)**, and SBR **(C)** values were substantially lower in the tumor--affected labyrinth compared with the unaffected contralateral labyrinth, and all interaural differences reached statistical significance (all *p* < 0.001).

We further analyzed a subgroup of 13 patients who underwent pre-contrast 3D-FLAIR imaging. Within this subset, CBR, VBR and SBR values were significantly elevated in the affected labyrinth compared with the intact contralateral side, and all interaural disparities reached statistical significance (all *p* < 0.001) (Figure 5). Specific values: affected side CBR (1.66 ± 0.60), unaffected side CBR (0.39 ± 0.18); affected side VBR (1.56 ± 0.67), unaffected side VBR (0.46 ± 0.21); affected side SBR (0.93 ± 0.36), unaffected side SBR (0.28 ± 0.14).

**Figure 5:**
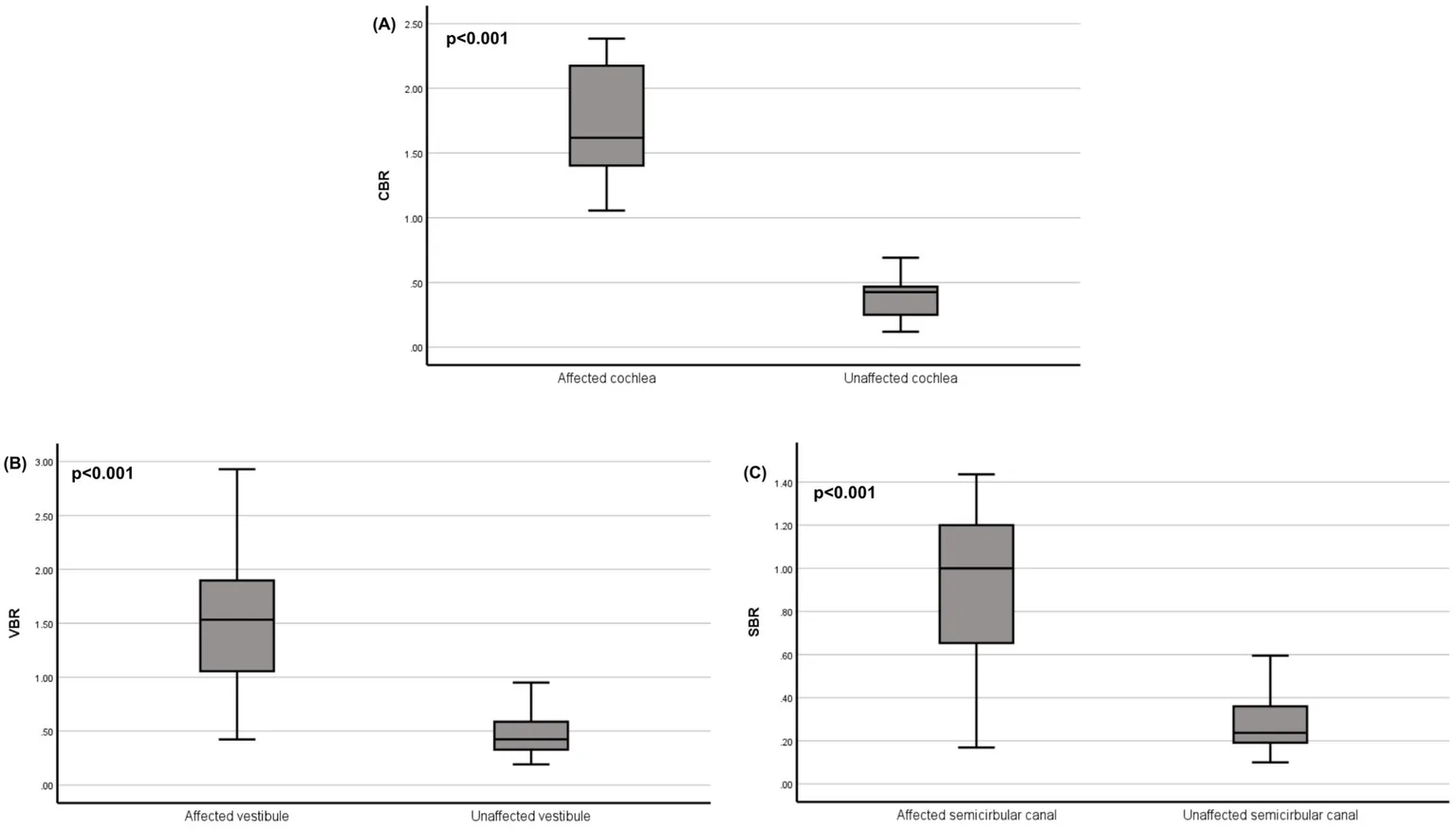
Quantitative signal ratios on pre-contrast 3D-FLAIR imaging. Significantly higher CBR **(A)**, VBR **(B)**, and SBR **(C)** values were observed in the tumor-affected labyrinth relative to the unaffected contralateral labyrinth, with all interaural differences statistically significant (all *p* < 0.001).

At the same time, visual qualitative evaluation of labyrinth signal was carried out. On delayed post-contrast 3D-FLAIR (*n* = 22), visually detectable hyperintensity was observed in the cochlea in 20/22 (90.9%) patients, in the vestibule in 15/22 (68.2%) patients, and in the semicircular canal in 8/22 (36.4%) patients. For the subgroup with pre-contrast 3D-FLAIR (*n* = 13), visually detectable hyperintensity was noted in the cochlea in 12/13 (92.3%), vestibule in 11/13 (84.6%), and semicircular canal in 9/13 (69.2%) of patients. It is worth noting that on the pre-contrast 3D-FLAIR sequence, the tumor-affected labyrinth usually shows iso-intensity compared with the brain stem, while the contralateral unaffected labyrinth usually shows hypointense. Therefore, when compared with the brain stem, the pathological signal changes on pre-contrast 3D-FLAIR often show iso-intensity, rather than significantly bright hyperintensity.

### 3.4 Correlation between multi-sequence MRI signal ratios and audiovestubular dysfunctions

No meaningful correlations were detected between affected-side CBR, VBR and SBR values and ipsilateral hearing impairment for both delayed postcontrast 3D-FLAIR and T2WI sequences. For pre-contrast 3D-FLAIR scans, however, CBR of the lesion ear presented a moderate positive association with hearing loss (*r* = 0.582, *p* = 0.037), whereas VBR and SBR failed to display statistically relevant links with auditory dysfunction (Table 2).

**Table 2:** Correlations between multi-sequence MRI signal ratios and audiometric outcomes.

MRI sequences	Signal ratios	PTA	
		Correlation coefficients (95% CI)	*p* value
Delayed postcontrast 3D-FLAIR	CBR	0.407 (−0.031, 0.713)	0.067
	VBR	0.203 (−0.251, 0.584)	0.377
	SBR	0.135 (−0.304, 0.527)	0.550
T2WI	CBR	−0.410 (−0.709, 0.014)	0.058
	VBR	0.087 (−0.347, 0.491)	0.699
	SBR	0.003 (−0.419, 0.424)	0.990
pre-contrast 3D-FLAIR	CBR	0.582 (0.045, 0.858)	0.037
	VBR	0.242 (−0.357, 0.700)	0.426
	SBR	0.478 (−0.099, 0.814)	0.098

PTA, pure-tone audiolometry; CI, confidence interval; CBR, cochlea-brainstem ratio; VBR, vestibule-brainstem ratio; SBR, semicircular canal-brainstem ratio.

The three inner ear signal ratios in the pre-contrast 3D-FLAIR and delayed post-contrast 3D-FLAIR showed no significant correlation with the caloric test results. In the T2WI sequence, only the SBR on the affected side was correlated with vestibular function abnormalities in the caloric test (*r* = 0.440, *p* = 0.040), while ipsilateral CBR and VBR values failed to yield any significant associations with caloric test outcomes (Table 3).

**Table 3:** Correlations between multi-sequence MRI Signal Ratios and vestibular function findings.

MRI sequences	Signal ratios	Caloric test		vHIT	
		Correlation coefficients (95% CI)	*p* value	Correlation coefficients (95% CI)	*p* value
Delayed postcontrast 3D-FLAIR	CBR^a^	0.087 (−0.347, 0.491)	0.699	0.396 (−0.031, 0.700)	0.068
	VBR^b^	0.017 (−0.418, 0.445)	0.970	0.246 (−0.209, 0.611)	0.267
	SBR^b^	0.173 (−0.268, 0.554)	0.449	0.039 (−0.380, 0.453)	0.858
T2WI	CBR^b^	0.055 (−0.375, 0.466)	0.802	0.079 (−0.354, 0.484)	0.747
	VBR^a^	−0.063 (−0.472, 0.369)	0.779	−0.200 (−0.573, 0.242)	0.371
	SBR^b^	0.440 (0.024, 0.728)	0.040	0.189 (−0.253, 0.565)	0.407
Pre-contrast 3D-FLAIR	CBR^a^	0.278 (−0.322, 0.719)	0.357	0.156 (−0.433, 0.651)	0.610
	VBR^a^	0.417 (−0.175, 0.787)	0.156	0.030 (−0.529, 0.571)	0.922
	SBR^a^	−0.061 (−0.592, 0.507)	0.842	0.123 (−0.459, 0.631)	0.689

a Effect sizes were computed by Pearson correlation for normally-distributed variable.

b Effect sizes were computed by Mann–Whitney U test for non-normally-distributed variable.

vHIT, video head impulse test; CI, confidence interval; CBR, cochlea-brainstem ratio; VBR, vestibule-brainstem ratio; SBR, semicircular canal-brainstem ratio.

Across all three MRI sequences, no statistically significant correlations between ipsilateral CBR, VBR or SBR measurements and high frequency vestibular function metrics derived from vHIT were detected within the present cohort (Table 3).

## 4 Discussion

While MRI serves as the diagnostic gold standard, current protocols primarily focus on tumor morphology rather than the subtle micro-environmental alterations within the adjacent labyrinth that precede functional deterioration. Given this context, clinicians still lack reliable non-invasive candidate imaging correlates to identify early breakdown of the blood-labyrinth barrier and derangements of inner ear fluid homeostasis (14, 15). To fill this clinical research gap, the present work systematically analyzed high-resolution multi-sequence MRI measurements in a cohort of VS patients, focusing on signal intensity ratios calculated from T2WI, pre-contrast 3D-FLAIR, and 4-h delayed postcontrast 3D-FLAIR scans. We then correlated these quantitative imaging readouts with complete audiovestibular test profiles to clarify whether such metrics could correlate with cochlear and vestibular functional status.

One core observation from our analysis was the stark, inverse pattern of signal changes seen across T2WI and 3D-FLAIR sequences, which mirror separate pathological manifestations of disrupted inner ear fluid homeostasis. The uniform depression of cochlear, vestibular, and semicircular canal signal on high-resolution T2WI in our tumor-side ears suggests a fundamental alteration in fluid composition. Pre-contrast 3D-FLAIR imaging, which suppresses pure fluid signals while retaining sensitivity to proteinaceous fluid, shows uniform hyperintensity throughout the entire inner ear. Hypointense signals on T2WI and hyperintense signals on pre-contrast 3D-FLAIR in tumor-bearing ears indicate fundamental changes in inner ear fluid composition, which are most likely attributable to elevated protein concentration or increased viscosity of the perilymph. The elevated signal intensity ratios on delayed post-contrast 3D-FLAIR provide direct evidence of pathologic blood-labyrinth barrier permeability. This characteristic imaging pattern consists of hypointensity on T2WI and hyperintensity on pre-contrast as well as delayed post-contrast 3D-FLAIR (16). Comprehensive analysis of this sign facilitates a more thorough understanding of the pathogenetic mechanisms responsible for labyrinthine lesions secondary to VS. The mass effect exerted by VS within the internal auditory canal obstructs axonal protein transport, induces blood-labyrinth barrier breakdown secondary to arteriovenous stasis, and triggers a cascade of aseptic inflammatory responses (17, 18). These processes ultimately result in protein extravasation into the perilymph and decompensation of endolymphatic homeostasis (19).

By analyzing the link between structural imaging findings and inner ear function, we found that imaging correlates from different MRI sequences can reliably predict inner ear dysfunction. Our analysis demonstrated that only the cochlear pre-contrast 3D-FLAIR signal intensity ratio had a significant positive correlation with hearing loss severity. No such meaningful correlation was seen for T2WI or delayed post-contrast 3D-FLAIR signal ratios. This unique relationship indicates that cochlear pre-contrast 3D-FLAIR hyperintensity better reflects subtle pathological changes including protein leakage and local inflammation, which directly damage hair cells and auditory neurons (20). By contrast, other signal metrics mainly reflect overall fluid composition or general blood-labyrinth barrier leakage and lack this specificity.

In contrast, the T2WI signal intensity ratio of the horizontal semicircular canal correlated significantly with caloric test asymmetry, whereas no such correlation existed for vHIT parameters. Such divergent imaging changes suggest that vestibular sensory structures adopt distinct pathological responses to tumor-induced damage. The caloric test evaluates vestibular function based on low-frequency thermal convection within the bony labyrinth, with its diagnostic efficacy primarily dependent on the physical properties and unobstructed flow of endolymph in the lateral semicircular canal (21). Accordingly, this test is highly susceptible to elevated endolymph viscosity, a pathological alteration that corresponds to decreased T2WI signal intensity on MRI (22). Notably, this functional imaging correlation has not been documented in prior VS research. This novel cross-sectional correlation highlights the potential research interest of these imaging metrics. Whether they can ultimately support screening for vestibular rehabilitation eligibility or fall-risk stratification requires further prospective clinical validation in larger cohorts (23). In comparison, the vHIT evaluates high-frequency vestibulo-ocular reflex function and barely relies on inner ear fluid movement. We found no statistically significant correlation between vHIT results and all three MRI signals in the present small cohort. This observation gives rise to the hypothesis that vestibular dysfunction detected by vHIT in patients with VS may mainly stem from direct nerve conduction impairment rather than labyrinthine fluid-circulation disorders (24). However, given limited statistical power, this hypothesis requires further validation in larger cohorts. These inconsistent correlations clearly show that multi-sequence MRI can separately characterize how VS damages different parts of the inner ear through distinct mechanisms. T2WI reflects pathological changes related to abnormal semicircular canal fluid, while pre-contrast 3D-FLAIR imaging detects protein accumulation lesions within the cochlea.

We observed obvious differences in delayed post-contrast 3D-FLAIR signals between the two ears, yet these signal values had no significant correlation with inner ear function. This indicates gadolinium contrast enhancement reflects widespread, non-specific blood-labyrinth barrier breakdown, and such leakage does not have a linear relationship with hearing or vestibular function thresholds. Whether such inconsistent findings are attributable to variations in disease duration requires further verification.

Several limitations of this study should be acknowledged. First, the modest overall sample size (*n* = 22), with an even smaller subgroup available for pre-contrast 3D-FLAIR analysis (*n* = 13), restricted statistical power and precluded rigorous subgroup comparisons as well as formal multivariable adjusted analyses accounting for potential confounders including age, tumor size, Koos grade, disease duration, and baseline hearing status. No multiple comparison correction was applied for exploratory correlation analyses. Nominal significant correlations from exploratory tests must be interpreted cautiously as hypothesis-generating findings and require external validation in larger cohorts. Only 13 patients had complete pre-contrast 3D-FLAIR sequences; this pattern of missing data may give rise to selection bias. Second, no prospective sample-size and power analysis was conducted. All patients complained of dizziness when they were admitted to the hospital, which may introduce selection bias, so we should be cautious when extending the results of this study to all patients with VS. In addition, Intra- and inter-observer reliability of manual ROI signal-ratio measurement was not evaluated in present study and requires dedicated validation before clinical application. Third, without histopathological confirmation, the exact biological mechanisms underlying the observed signal alterations—including potential protein exudation, inflammatory infiltration, or fibrous remodeling—remain speculative. In addition, the cross-sectional nature of this study prevents definitive inference of causality and limits our ability to characterize the temporal progression of imaging abnormalities and subsequent functional deterioration. Prospective multicenter studies with larger patient cohorts and correlative histopathological evidence are therefore warranted to validate these candidate imaging correlates and consolidate their prognostic value.

In summary, the present study demonstrates that quantitative signal intensity ratios derived from T2WI, pre-contrast 3D-FLAIR, and delayed post-contrast 3D-FLAIR can serve as sensitive, non-invasive candidate imaging correlates for the detection of unilateral inner ear pathology in VS. Notably, distinct sequence-specific functional correlations were observed: increased pre-contrast cochlea-to-brainstem signal ratios were moderately associated with hearing impairment, likely reflecting intralabyrinthine inflammatory and protein-rich pathological changes, whereas decreased T2WI signal ratios in the semicircular canals were linked to low-frequency vestibular dysfunction, suggestive of structural and hydrodynamic impairment. These findings highlight the complementary pathophysiological information provided by different MRI sequences. Whether these metrics can advance conventional anatomical imaging toward functional and risk-based stratification remains to be confirmed. The potential value of incorporating these quantitative imaging metrics into clinical workflows for early functional risk evaluation, supporting personalized treatment strategies or serving as imaging endpoints for monitoring disease progression requires further prospective external validation.

## Data Availability

The datasets presented in this study can be found in online repositories. The names of the repository/repositories and accession number(s) can be found in the article/Supplementary material.
