# Supplementary material for: Multi-sequence MRI signal intensity ratios as candidate imaging correlates for audiovestibular dysfunction in vestibular schwannoma
Source: Front Neurol. 2026 Sep 23;17:1936823. doi: 10.3389/fneur.2026.1936823 (PMC13642135; doi:10.3389/fneur.2026.1936823)
Supplement: Supplementary file 1 [file Table_4.DOCX]

Supplementary Table Koos grade subgroup comparisons

| MRI sequences | Signal ratios | Koos II | | P value |
| --- | --- | --- | --- | --- |
|  |  | Number | Number |  |
| Delayed postcontrast 3D-FLAIR | CBR | 11 | 10 | 0.978 |
|  | VBR | 11 | 9 | 0.681 |
|  | SBR | 11 | 10 | 0.503 |
| T2WI | CBR | 11 | 10 | 0.260 |
|  | VBR | 11 | 10 | 0.971 |
|  | SBR | 11 | 10 | 1 |
| pre-contrast 3D-FLAIR | CBR | 7 | 5 | 0.864 |
|  | VBR | 7 | 5 | 0.914 |
|  | SBR | 7 | 5 | 0.737 |

CBR, cochlea-brainstem ratio; VBR, vestibule-brainstem ratio; SBR, semicircular canal-brainstem ratio.
