# Supplementary material for: Multi-sequence MRI signal intensity ratios as candidate imaging correlates for audiovestibular dysfunction in vestibular schwannoma
Source: Front Neurol. 2026 Sep 23;17:1936823. doi: 10.3389/fneur.2026.1936823 (PMC13642135; doi:10.3389/fneur.2026.1936823)
Supplement: Supplementary file 2 [file Table_5.DOCX]

| **Item** | **Item Number** | **STROBE Recommendation** | **Section in revised manuscript** | **Remarks** |
| --- | --- | --- | --- | --- |
| Title and abstract | 1a | Indicate the study’s design with a commonly used term in the title or the abstract | Abstract-Methods | Retrospective observational study |
|  | 1b | Provide in the abstract an informative and balanced summary of what was done and what was found | Abstract full text | Background‑Methods‑Results‑Conclusions |
| **Introduction** |  |  |  |  |
| Background/  rationale | 2 | Explain the scientific background and rationale for the investigation being reported | Introduction | Gap: Lack of non‑invasive inner ear imaging correlates for VS audiovestibular impairment |
| Objectives | 3 | State specific objectives, including any prespecified hypotheses | Introduction | Primary hypothesis: inter ear difference of MRI signal ratios; correlation analyses defined as exploratory |
| **Methods** |  |  |  |  |
| Study design | 4 | Present key elements of study design early in the paper | Materials and Methods-Study Subjects | Retrospective single center cross‑sectional observational study |
| Setting | 5 | Describe the setting, locations, and relevant dates, including periods of recruitment and data collection | Materials and Methods‑Study Subjects | Setting: Shandong Provincial Hospital; enrolment November 2021 to February 2026 |
| Participants | 6a | Give the eligibility criteria, and the sources and methods of selection of participants. | Materials and Methods ‑Study Subjects  Figure 1 (participant flow chart) | inclusion/exclusion criteria listed; flow chart documents screening |
|  | 6b | Describe matching criteria (if any) | NA | No matching performed |
| Variables | 7 | Clearly define all outcomes, exposures, predictors, potential confounders, and effect modifiers. Give diagnostic criteria, if applicable | Materials and Methods- Image Post-processing and Analysis;  Materials and Methods-Audiometric and vestibular tests | Predictors: CBR/VBR/SBR;  Outcomes: PTA, caloric test, vHIT; confounders: age, tumor size, Koos grade, disease duration |
| Data sources/ measurement | 8 | For each variable of interest, give sources of data and details of methods of assessment (measurement). Describe comparability of assessment methods if groups are compared | Materials and Methods | 3.0T MRI parameters;  ROI manual‑measurement by blinded radiologist;  pure‑tone audiometry  caloric test (Jongkees’ formula provided), vHIT protocols |
| Bias | 9 | Describe any efforts to address potential sources of bias | Materials and Methods- Image Post-processing and Analysis; Discussion‑Limitations; | Radiologist blinded to functional results; Limitations section addresses selection bias, missing data, small sample bias |
| Study size | 10 | Explain how the study size was arrived at | Materials and Methods -Study Subjects; Discussion‑Limitations; | No prospective power/sample‑size calculation; all complete‑dataset eligible retrospective cases were included |
| Quantitative variables | 11 | Explain how quantitative variables were handled in analyses. If applicable, describe which groupings were chosen and why | Materials and Methods-Statistical Analysis; | Continuous MRI‑ratios, PTA; caloric / vHIT dichotomized as normal‑abnormal; normality testing reported; different correlation analysis methods were selected according to whether the data were normally distributed |
| Statistical methods | 12a | Describe all statistical methods, including those used to control for confounding | Materials and Methods-Statistical Analysis; | Paired‑t / Wilcoxon signed‑rank; Spearman rank correlation; Pearson correlation or Mann-Whitney U test |
|  | 12b | Describe any methods used to examine subgroups and interactions | Supplementary file 5-Koos grade subgroup comparisons  Discussion‑Limitations; | Exploratory Koos grade I vs II subgroup comparison in supplementary material; no formal interaction analysis |
|  | 12c | Explain how missing data were addressed | Results‑Baseline characteristics;  Discussion‑Limitations; | No data imputation; Pre‑contrast‑3D‑FLAIR only available for n=13 subset; analyses restricted to available cases |
|  | 12d | If applicable, describe analytical methods taking account of sampling strategy | NA | No complex sampling strategy applied |
|  | 12e | Describe any sensitivity analyses | NA | No pre‑specified sensitivity analyses performed; all correlation results explicitly labelled exploratory |
| **Results** |  |  |  |  |
| Participants | 13a | Report numbers of individuals at each stage of study: potentially eligible, examined eligibility, confirmed eligible, included in study, analyzed | Results‑Baseline characteristics;  Figure 1 (participant flow chart) | Flow‑chart documents screening, exclusion reasons, final n=22; pre‑contrast‑3D‑FLAIR subset n=13 clearly marked |
|  | 13b | Give reasons for non‑participation at each stage | Figure1 legend; | Diagnosis of VS was inconclusive |
|  | 13c | Report characteristics of participants with missing data, compared to participants without missing data | Results‑Baseline characteristics;  Discussion‑Limitations; | Missing pre‑contrast‑3D‑FLAIR from protocol change, not patient characteristics; potential selection bias discussed |
| Descriptive data | 14a | Report descriptive data for participants: demographic, clinical, exposure‑related variables | Table 1 | Age, gender, tumor side, tumor size, Koos grade, PTA, caloric / vHIT results |
|  | 14b | Report numbers of participants with missing data for each variable of interest | Results‑Baseline characteristics;  Discussion‑Limitations; | Pre‑contrast 3D‑FLAIR missing for 9/22 patients |
|  | 14c | Cohort study: Summarize follow-up time—e.g., average and total amount | NA |  |
| Outcome data | 15 | Cross‑sectional specific: Report numbers of outcome events or summary measures | Results‑Audiological and Vestibular Function Test Results;  Results‑Inner Ear MRI Signal Ratios between VS-affected and contralateral unaffected ears | PTA mean ±SD; number of abnormal caloric test (16/22); abnormal vHIT (6/22); number of pathological semicircular canals, catch‑up saccades reported  Inner Ear MRI Signal Ratios |
| Main results | 16a | Give unadjusted estimates and, if applicable, confounder‑adjusted estimates and their precision (e.g., 95% confidence intervals). Make clear which confounders were adjusted for and why they were included. | Results-Correlation between multi-sequence MRI Signal Ratios and audiovestubular dysfunctions; | Effect‑sizes and 95%CI added for correlations and paired comparisons; no multivariate adjusted estimates (small sample limitation) |
|  | 16b | Report category boundaries when continuous variables were categorized | Materials and Methods-Audiometric and vestibular tests | Caloric canal paresis >25%; vHIT gain thresholds defined |
|  | 16c | If relevant, consider translating estimates of relative risk into absolute risk for a meaningful time period | NA | Not applicable for this imaging‑functional correlation study |
| Other analyses | 17 | Report other analyses done: subgroup‑analyses, interaction‑analyses, sensitivity‑analyses | Supplementary file 5-Koos grade subgroup comparisons  Discussion‑Limitations; | Exploratory Koos‑grade subgroup comparison, labelled exploratory |
| **Discussion** |  |  |  |  |
| Key results | 18 | Summarise key results with reference to study objectives | Discussion opening paragraph; | Match primary hypothesis and exploratory correlation findings |
| Limitations | 19 | Discuss limitations of the study, taking into account sources of potential bias or imprecision | Discussion‑Limitations; | Small sample, no multiple‑comparison correction, no inter‑/intra‑observer reliability, selection bias, lack of non‑VS control group, no predictive modelling |
| Interpretation | 20 | Discuss results in context of existing evidence; interpret results cautiously | Discussion full text | Compare to prior VS inner‑ear MRI literature, the potential for MRI metrics to guide personalized management strategies requires further prospective external validation |
| Generalisability | 21 | Discuss generalisability (external validity) of the findings | Discussion‑Limitations | Only dizzy VS patients enrolled; cannot generalise to incidental asymptomatic VS cohort |
| **Other information** |  |  |  |  |
| Funding | 22 | Give sources of funding and role of funders | Funding section | No funding declared |
